# Supplementary material for: Blood Neurofilament Light Chain and Glial Fibrillary Acidic Protein as Promising Screening Biomarkers for Brain Metastases in Patients with Lung Cancer
Source: Int J Mol Sci. 2024 Jun 10;25(12):6397. doi: 10.3390/ijms25126397 (PMC11204234; doi:10.3390/ijms25126397)
Supplement: Supplementary file 1 [file ijms-25-06397-s001.zip › ijms-3007640-supplementary.pdf]

Supplementary Figure S1. Nomogram for predicting brain metastasis in patients with advanced lung cancer (stage III-IV) (n=379) : (A) Study design, (B) Predictive accuracy, and (C) Calibration curves with Hosmer–Lemeshow test.

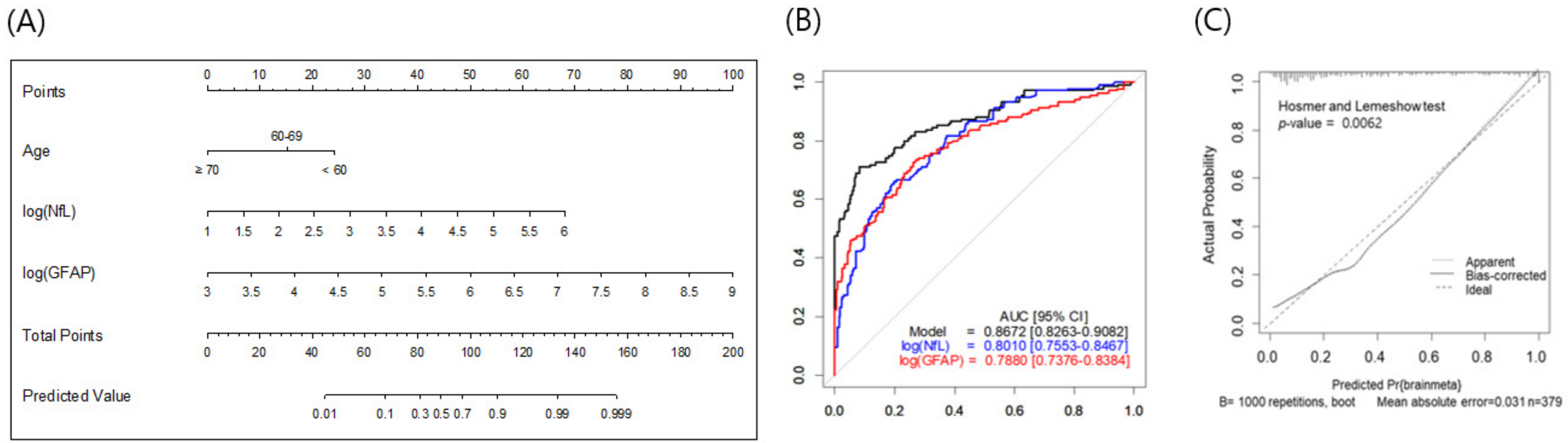

Abbreviations: NfL, serum neurofilament light chain; GFAP, glial fibrillary acidic protein

Supplementary table S1. Univariable and multivariable logistic regression to predict brain metastases in patients with stage III-IV lung cancer (N=379)

		**Univariable**		**Multivariable**	
		**OR (95% CI)**	**P-value**	**OR (95% CI)**	**P-value**
**Age**					
	<60 years	1 (ref)	**(0.0079)**	1 (ref)	**(<.0001)**
	60-69 years	0.724 (0.439-1.194)	0.2053	0.397 (0.201-0.787)	0.0082
	≥70 years	0.429 (0.251-0.731)	0.0019	0.082 (0.036-0.185)	<0.0001
**Sex**					
	Male	1 (ref)			
	Female	1.066 (0.687-1.653)	0.7758		
**BMI**					
	≤25	1 (ref)			
	>25	0.472 (0.292-0.764)	**0.0022**		
**Smoking**					
	Current smoking	1 (ref)	(0.4192)		
	Previous smoking	0.752 (0.418-1.351)	0.34		
	Never smoked	1.043 (0.616-1.767)	0.8753		
**HTN**					
	Non-HTN	1 (ref)			
	HTN	0.449 (0.238-0.847)	**0.0134**		
**DM**					
	Non-DM	1 (ref)			
	DM	0.762 (0.418-1.387)	0.3732		
**Histology**					
	ADC	1 (ref)			
	SCC	0.960 (0.550-1.675)	0.8855		
**log(sNfL)**					
		4.686 (3.300-6.654)	**<0.0001**	4.091 (2.600-6.439)	**<0.0001**
**log(sGFAP)**					
		7.200 (4.415-11.743)	**<0.0001**	5.608 (3.038-10.352)	**<0.0001**

Abbreviations: DM, diabetes mellitus; HTN, hypertension; BMI, body mass index; sNfL, serum neurofilament light chain; sGFAP, serum glial fibrillary acidic protein; ADC, adenocarcinoma; SCLC, small cell lung cancer; CI, confidence interval
